# Clinimetric quality of the fire fighting simulation test as part of the Dutch fire fighters Workers' Health Surveillance

**DOI:** 10.1186/1472-6963-10-32

**Published:** 2010-02-04

**Authors:** Marie-Christine J Plat, Monique HW Frings-Dresen, Judith K Sluiter

**Affiliations:** 1 Academic Medical Center, University of Amsterdam, Department: Coronel Institute of Occupational Health, Amsterdam, the Netherlands

## Abstract

**Background:**

Clinimetric data for the fire fighting simulation test (FFST), a new test proposed for the Workers' Health Surveillance (WHS) of Dutch fire fighters, were evaluated.

**Methods:**

Twenty-one fire fighters took the FFST three times with one and three weeks between testing. Clinimetric quality was determined by means of reliability, agreement and validity. For reliability and agreement, the intraclass correlation coefficient (ICC), and standard error of measurement (SEM), were analysed. For construct validity, the tests from 45 fire fighters were correlated with their own and their supervisors' rated work ability.

**Results:**

The ICCs were 0.56 and 0.79 at the one-week and three-week test-retest periods, respectively. Testing times ranged from 9 to 17 minutes; the SEMs were 70 s at the one-week and 40 s at the three-week test-retest periods. The construct validity was moderate (-0.47 ≤ r ≤ -0.33; p < 0.05).

**Conclusions:**

The FFST was reliable with acceptable agreement after three weeks. Construct validity was moderate. We recommend using FFST as a part of the WHS for Dutch fire fighters. It is advised that fire fighters should perform the FFST once as a trial before judging their performance in testing time during the second performance.

## Background

In 1998, the International Labour Organization described the principle behind the workers' health surveillance (WHS) as being the 'primary prevention of occupational and work-related diseases and injuries'. They also suggested that data collected in the WHS should be applied to protect the health of employees [1]. The WHS should focus on signaling emerging work-related risk factors and work-related health complaints; additionally, it should result in the application of relevant interventions to prevent decreased work ability. In jobs with specific job demands, the WHS is of special importance. Specific job demands are comprised of elicited exposures that cannot be prevented or may reveal safety risks at the workplace [2]. The employee's health and safety and, in some occupations, that of third persons may be in danger if the occupational health requirements of the worker no longer fit the specific demands of a job. An occupation that has such specific occupational health requirements is fire fighting.

Previous studies have shown that fire fighting is a demanding job, both physically and mentally. The fact that fire fighting is physically demanding is described in several studies that have shown that fire fighters have high energetic and biomechanical workloads, as well as requirement of postural and movement control during their job activities [3-8].

A periodic WHS for Dutch fire fighters was developed by Sluiter and Frings-Dresen in 2006 and physical, physiological and mental demands, as well as cardiovascular risk factors, formed the basic principles for its contents [9]. When determining work ability in jobs with specific demands, Sluiter [10] described performance measures that should be included and assessed with regard to those specific job demands. Specific job demands in fire fighting are, for example, the ability to clamber and climb as well as the ability to lift and drag heavy loads.

Because physically high demanding tasks are a part of the job, physical performance tests are a part of the surveillance [8,9,11]. The physical tests, therefore, should be based on real job activities. Recently, job-specific tests have been used for male fire fighters [12], candidate fire fighters [13], in the WHS of Dutch ambulance workers [10] and for beach lifeguards [14].

The Dutch WHS for fire fighters consists, among other things, of two job-specific physical tests: a fire fighting stair climb test and the fire fighting simulation test (FFST). The FFST is based on a Canadian test developed by Deakin et al. [15] The test from Deakin consisted of the simulation of ten fire fighting tasks, such as a hose carry, a ladder climb, a hose pull, a forcible entry task and a victim drag. This test was adapted for the Dutch situation in the fire department of Rotterdam [16], but it needed further testing before practical application was found to be feasible.

Before implementation of the test as part of the WHS clinimetric characteristics of the test, such as reproducibility, i.e., reliability and agreement, and construct validity, should be determined [17]. Reliability is important when a test is used to discriminate between individuals, as reliability is the ability of a test to distinguish persons despite measurement error. In the case of an evaluative instrument, agreement parameters are also required. Agreement reflects the to-be-expected (extent of the) differences when assessed in repeated measurements scores [18] i.e., the level of agreement of a score at the first testing moment compared to a score at the second testing moment for the same person. Agreement is a property of the test, and it should therefore be tested in the population in which it will be used [19]. Because the FFST will be used in the future for discriminative and evaluative purposes, both clinimetric qualities are relevant.

In addition to the reproducibility, it is important to know whether the test measures what it intends to measure: therefore, the validity should be determined. The construct of 'work ability of fire fighters tested during job-simulated activities' is examined in the FFST, and its construct validity should be determined [20]. To examine the construct validity of the test, it should be compared with a gold standard for measuring the workability of fire fighters. However, as no gold standard is present at this moment, a composite reference standard of related variables [21] to test the convergent validity, as part of the construct validity, can be used.

As the test is not yet used in a large population of fire fighters in the Netherlands, the exact scoring method has not yet been developed and stipulated. Time was used as a primary scoring criterion in the original test and in practice [15]. Therefore, time of executing the FFST will be used for determining the clinimetric quality.

The aforementioned clinimetric properties are not yet determined for the FFST. Therefore, we evaluated the reliability, agreement and construct validity of the fire fighting simulation test in a population of Dutch fire fighters in this study.

## Methods

### Subjects

Three regional fire departments throughout the Netherlands were involved in this study. In each department, a random sample of fire fighters was invited to execute the FFST after receiving information about the study. Both volunteer and professional fire fighters were invited, and all subjects provided written informed consent. The study was performed in accordance with the Declaration of Helsinki and was approved by the ethics committee of the Academic Medical Center.

### Subjects reliability and agreement

A random sample of 21 fire fighters executed the FFST three times to test reliability and agreement. However, one participant's time of execution was not registered at the third testing moment; therefore, results for this participant were excluded from every testing moment. Additionally, one participant's scores changed in an extraordinary way compared to other participants; therefore, this outlier was also excluded. The results of 19 participants were used for the final analysis of the reliability and agreement. All participants were male and carried out operative tasks. Sixteen participants were professionals, and three were volunteer fire fighters. The participants' mean age was 35 years (SD 9; range 21-52), their mean body weight was 86 kg (SD 11; range 74-112) and their mean height was 182 cm (SD 5; range 170-189).

### Subjects construct validity

To test construct validity a total of 45 professional fire fighters (43 males, 2 females) from one region performed the FFST once. Data from five of the participants were also used in the reproducibility part of the study. All participants carried out operative tasks. The participants' mean age was 38 years (SD 9; range 24-54), their mean body weight was 87 kg (SD 10; range 67-112) and their mean height was 182 cm (SD 6; range 172-198).

### Fire fighting simulation test

**Figure 1:**
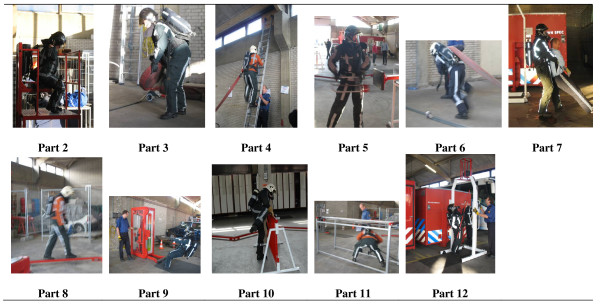
**Each consecutive part of the fire fighting simulation test (except part 1 of the fire fighting simulation test, getting ready for turn-out)**.

The fire fighting simulation test is a simulation of daily consecutive fire fighting activities. The whole test takes between about 10 to 15 minutes. An extensive description of the FFST can be found in Table 1; for photographs of the FFST, see Figure 1. 

The test contained 12 parts that were successively executed:

**Table 1:** Description of the parts of the fire fighting simulation test.

Part of the FFST	Explanation of the part
1) Getting ready for turn-out	Subject is waiting in station clothes (blouse and pants) for the starting sign. After the starting sign, participants put on standard fire fighter turn-out gear and boots and walk 15 meters to point two.

2) Attaching SCBA, putting on gloves and carrying two hoses	Attach the SCBA and mask, which are ready at a fire engine (or simulation platform) on the place of number 1. Put on gloves, afterwards take two 52-mm hoses and walk 15 meters to point three.

3) Throwing, coupling and dragging hoses	Put down one hose, throw the second hose to a point 15 meters ahead, take the connection of one hose, and afterwards walk to the end with one end of the hose. Couple two hoses and walk with one end of the hose to the start of point three; afterwards, walk 17 meters to four.

4) Setting up ladder, climbing ladder three times to the 10^th ^rung with fire fighting gear	Ladder stands straight up to the wall, put ladder in a good position, and slide until the tenth marked rung. Twist the rope around the 3^rd ^and 5^th ^rung and make a knot at the 4^th ^rung. Take the tool box from the fire engine, line and spout. Walk back to the ladder and walk up and down to the 10^th ^rung with the fire fighting gear, one after the other. Walk 15 meters to point five.

5) Connecting SCBA and forcible entry simulating hitting a resistance	Walk from point five to the fire fighting engine (15 m), take the sledge hammer there, and walk back to point five. Read and mention the amount of air, connect the SCBA, and hit the sledge hammer against the resistance. Move the resistance over 30 cm, the instructor tells when it moved 30 cm. Walk 15 meters to six.

6) Dragging hose filled with water	A 75-mm hose, half-filled with water and ending with a spout, lies zigzag near the fire engine. Take hose over shoulder and stretch forwards to the end, 15 meters. Afterwards walk 19 meters to point seven.

7) Rescuing dummy	Take the dummy in a grip, according to Rautek, and drag the 80-kg dummy 15 meters backwards and turn around and walk again 15 meters backwards to the starting point. Attention is paid to the manner of exertion from the legs, with straightened back. Walk 15 m to eight.

8) Walking a balance beam	Four beams lie down in a zigzag. Walk over a balance beam. If falling off, start again. Walk 15 m to nine.

9) Hose dragging simulation	Drag hoses two times to a distance of 15 meters. The apparatus simulates dragging a hose. Firstly, drag the hose 15 meters, walk around a counter, and once more drag the hose 15 meters. Walk 15 m to point ten.

10) Stepping/climbing over a fence	Step/climb over a fence of 1.03 m (not jumping) and walk 15 m to point eleven.

11) Smoke dive simulation with hose, standing and squatting.	Take a high-pressure hose forwards and backwards over 15 meters: 3 meters walking forward, 3 meters under tunnel (height 1.20 m) walking squatted, 3 meters normally, 3 meters under tunnel squatted and 3 meters normally, all forwards and subsequently the same way backwards. Walk 13 m to the last component, twelve.

12) Ceiling demolition simulation	Simulate demolishing the ceiling by knocking a heavy ball with a massive bar, with the ball hanging out of the ceiling. Let the ball touch ten times the top side of the basket. The instructor is counting aloud.

1) Getting ready for turn-out, putting on fire fighter turnout gear; 2) attaching self contained breathing apparatus (SCBA) in the fire engine, putting on gloves, getting out of the fire engine and taking two hoses; 3) throwing one hose, walking, throwing the second hose, coupling hoses and dragging the hose; 4) setting up a ladder, climbing the ladder three times with fire fighting gear; 5) connecting the breathing apparatus and hitting a resistance; 6) dragging a hose filled with water; 7) rescuing a dummy; 8) walking across a beam; 9) hose dragging simulation; 10) stepping/climbing over a fence; 11) smoke dive simulation with hose, in both the standing and squatting position; 12) ceiling demolition simulation [15,16].

Before performing the FFST, all participants watched the instructional DVD of the FFST in which all 12 parts of the test and their order were explained and demonstrated. This is technically similar to how fire fighters learned those skills. Before participants started the FFST, they were instructed to perform as quickly as possible, within the participants' abilities. Two instructors, a sports and technical fire instructor, accompanied each participant during the test (to optimise the execution of the test and to lead participants to the next part and monitor their performance for safety reasons), but they were told to not encourage the fire fighters verbally during the test.

During the first part, participants were asked to put on their own protective clothing (including pants, coats, helmet, boots and gloves) and the SCBA was worn for the second part. The protective clothes and SCBA weighed 21 kg altogether. For parts five and above, tasks were executed while connected to the SCBA.

The FFST was scored by fire fighting sports instructors and technical fire instructors, and the results for the testing time needed to complete the test (in seconds) were recorded in a structured results table. The instructors could decide to stop the test if the participant was thought to be in physical danger by continuing the test (e.g., feeling too dizzy to continue the FFST). Nearly all fire fighters performed the FFST for the first time, and two of the fire fighters had performed the FFST once before this study.

### Procedure

Before starting the FFST, participants filled out the Physical Activity Readiness Questionnaire (PAR-Q) [22]. The PAR-Q provides an indication about safety by which someone could energetically perform demanding tests. If one of the questions was positive, the participant received face-to-face contact with the occupational physician (OP) before executing the test. Based on that interaction, the OP decided directly whether the participant could perform the test and whether the OP should be present during the test.

### Reliability, agreement and test-retest moments

**Figure 2:**
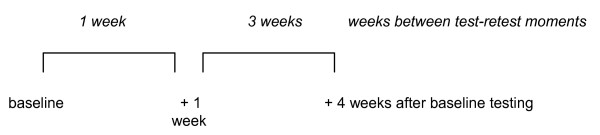
**Test-retest moments**.

A within-subjects design was used to test reliability and agreement. Fire fighters were tested a total of three times: the first time at baseline, the second time one week after baseline, and the third time four weeks after baseline testing. The first and second performances were compared (one week in between), and the second and third performances were compared (three weeks in between). A graphic of the test-retest moments is shown in Figure 2. Test-retest periods varied between five to ten days for the one-week period and between 20 to 28 days for the three-week period. The performances of the participants were standardised at daytime (between 8 and 17 o'clock).

### Construct validity

To examine the convergent validity as a part of construct validity, the testing time of the FFST was compared with separate variables and a composite reference standard of related variables [21]. First, the fire fighter filled in two items of the Work Ability Index (WAI) [23], adjusted for fire fighting work. Fire fighters scored their own current work ability on an 11-point scale, in which '0' was completely unable to work and '10' referred to work ability at its best in executing fire fighting tasks (first item of the WAI). In addition, fire fighters scored their work ability for executing fire fighting tasks in relation to the physical demands. Responses were given on a 5-point scale varying from very poor (1) to very good (5). For the composite reference standard, two variables, work ability and work ability with respect to physical demands (both self-reported), were summed. The sum total for this composite reference test ranged between 1 and 15. These data were collected before the first FFST. Second, the judgement of the direct supervisor on the current work ability of fire fighters was requested, similar to the first WAI item described above. Two fire fighter supervisors from one fire department judged 45 fire fighters together, and, the judgements of the supervisors were collected after the first test.

### Statistical analysis

SPSS 16.0 was used to perform the statistical analysis. Reliability and agreement were calculated by using the testing time (in seconds). Means, standard deviations and ranges of the testing time were calculated.

To determine reliability, the intraclass correlation coefficient (ICC) was calculated by using the ICC model 2.1 A, random two-way analysis, according to Shrout and Fleiss [24]. The ICC between baseline and after one week was calculated, as well as the ICC between one week and three weeks thereafter. An ICC with a value of <0.70 was considered as low reliability and ≥0.70 as high, as this classification was described for health outcomes [25]. To determine the degree of agreement, the standard error of measurement (SEM) was calculated by means of components of variance (SEM = √ (σ^2 ^testing moments + σ^2 ^error) [18]. To visualise the degree of agreement, a Bland and Altman plot with 95% limits of agreement was plotted [26]; the difference between two testing moments was calculated, and limits are shown in the plot in which 95% of the differences fall.

For the convergent validity, the Spearman's rank correlation coefficient between the outcome of the test (testing time) and the judgment of work ability was determined. This correlation coefficient was calculated between *i*) FFST testing time and the participants' own judgment of their work ability and *ii*) FFST testing time and the supervisors' judgment of the work ability of the participant. Convergent validity correlation was considered to be low if r < 0.30; moderate if 0.30 ≤ r < 0.60 and good if r ≥ 0.60 [27].

## Results

### Reliability and agreement

The means, standard deviations and ranges for the three testing moments, as well as the ICCs with 95% confidence intervals are presented in Table 2. On average, fire fighters performed the first test moment in 13.8 minutes (828 s) and the second test moment in 12.6 minutes (754 s). Compared with the second test moment, the average time of the third test moment was 12.6 minutes (756 s). Differences in mean testing time between baseline, one week and three weeks are present. The single measure ICC between baseline and one week was 0.56, and between one week and one month, it was 0.79.

**Table 2:** FFST testing time (s) for the different testing moments with reliability levels.

	*First measurement of FFST*				*Second measurement of FFST*				*ICC*	*ICC 95% CI*	
				
Testing moments compared	N	Mean	SD	Range	N	Mean	SD	Range		Lower	Upper
One-week test-retest moment	19	828	89	676-977	19	754	95	600-919	0.56	-0.43	0.83
Three-week test-retest moment	19	754	95	600-919	19	756	79	539-896	0.79	0.53	0.91

The agreement, expressed as the standard error of measurement (SEM), is 70 seconds for the one-week test-retest moment and 40 seconds for the three-week test-retest period, as can be seen in Table 3.

**Table 3:** Components of variance and measurements of agreement.

FFST testing moments compared	N	*Variance persons*	*Variance testing moments*	*Variance error*	*SEM (s)*
One week	19	6151.0	2572.4	2336.4	70.1
Three weeks	19	5971.9	0	1580.9	39.8

**Figure 3:**
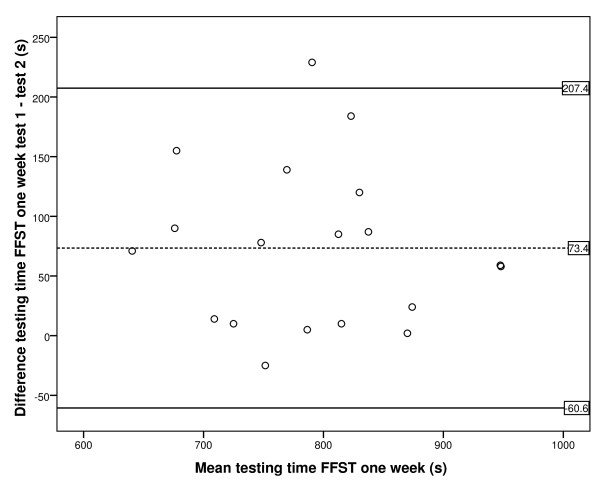
**Bland and Altman plot with 95% limits of agreement for FFST at the one-week retest**. Dotted line is the mean difference between FFST testing time for moments one and two. Continuous lines represent the 95% limits of agreement.

**Figure 4:**
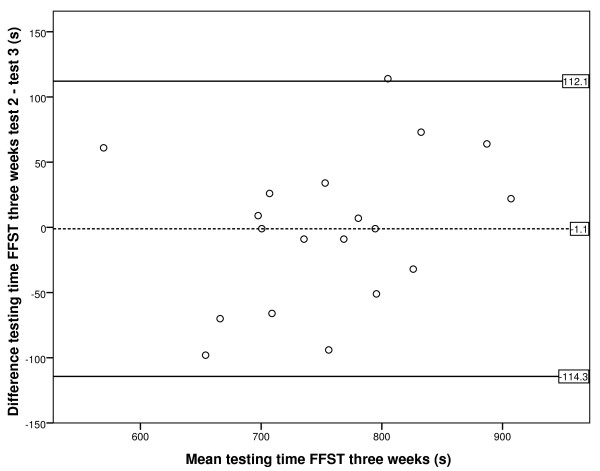
**Bland and Altman plot with 95% limits of agreement for FFST at the three-week retest**. Dotted line is the mean difference between FFST testing time for moments two and three. Continuous lines represent the 95% limits of agreement.

Figures 3 and 4 show the Bland Altman plot of the difference in testing time of the two performances of the FFST; the 95% limits of agreement are also illustrated. Each point represents one participant. For the one-week test-retest period, Figure 3 shows the 95% limits of agreement to be -61 s to 207 s. The 95% limits of agreement for the three-week test-retest period were -114 s to 112 s, as can be seen in Figure 4.

### Construct validity

The medians of the self-reported and supervisors-reported scores are given in Table 4. As can be seen, all medians of the reported scores on work ability are relatively high.

**Table 4:** Description of the work ability scores (N = 45).

	N	*Median*	*Interquartile range (Q3-Q1)*
Work ability (0-10)	45	8.0	1.8
Physical demands work ability (1-5)	45	4.0	1.5
Work ability and physical demands (1-15)	45	12.0	2.0
Work ability by supervisors (0-10)	45	7.0	1.0

Correlation coefficients between the work ability scores and testing time of the FFST are presented in Table 5. Outcomes showed moderate levels of convergent validity. The correlation coefficient between FFST testing time and self-rated scores of 1) work ability, 2) physical demands of the work ability and 3) work ability combined with physical demands were significant in both groups and ranged from -0.30 to -0.60. The higher the rating of work ability, the faster the testing time. The correlation coefficients between testing times and supervisors-rated work ability scores were also of moderate level and were significant (Table 5).

**Table 5:** Correlations (N = 45) between FFST testing time and different scores on work ability.

	N	*FFST testing time Spearman's rho*	*P*
Work ability	45	-0.42	0.004
Physical demands work ability	45	-0.34	0.021
Work ability + physical demands	45	-0.47	0.001
Work ability rated by supervisors	45	-0.33	0.026

## Discussion

The aim of this study was to evaluate the reproducibility, i.e., reliability and agreement, and the construct validity of the FFST as a performance-based, job-specific test proposed for use in the WHS for Dutch fire fighters. The reliability of the FFST was 0.56 for the one-week test-retest period and was 0.79 for the three-week test-retest period, indicating high reliability. The agreement was 70 s after one week, but 40 s after three weeks. The correlation coefficients to test the convergent validity varied slightly and were all of moderate levels.

The job-specific physical test was studied for clinimetric quality in this study, as part of the WHS for fire fighters. The WHS for fire fighters also included the fire fighter stairclimb test, as well as measurements to determine the mental effects of the job, general health aspects, chronic diseases and cardiovascular risk factors. After completion of the WHS, an occupational physician studied the results of the fire fighters and gave feedback individually in a face-to-face meeting. During this meeting, the occupational physician could begin or advice interventions when required by the WHS protocol.

With the interventions and advise of the OP fire fighters' decreased job functioning will be prevented. The health of fire fighters will become better, and they should be able to perform their public tasks better. Third parties, e.g. the public and colleagues, will therefore be safer if the fire fighters' performances are safeguarded.

### Reliability

Reliability is the ability of a test to distinguish persons despite measurement error and depends on the ratio between the ranges of attained scores versus measurement error [18]. In our study, the variation in testing outcome of the FFST testing time is not an infinite number. Testing times ranged from 9 to 17 minutes, and testing times could not be expected to be shorter due to the nature of the test, which consisted of twelve parts, all of which had to be executed. From a clinimetric standpoint, the calculated ICC of 0.79 at a three-week test-retest period could be interpreted as high compared to the magnitude of the range; consequently, we believe the FFST can be considered reliable and it should be therefore used in practice for discriminative purposes.

The FFST is a new test, the reproducibility of which has never been tested before. The original Canadian fire fighter test of Deakin et al. [15] was tested on test-retest reliability (with a test-retest period of one day) with a high correlation coefficient (r = 0.93). Nevertheless, the original test from Canada was adapted to the specific Dutch fire fighter situation by van Blitterswijk et al. [16], and some parts were changed. Three parts were excluded in the Dutch test compared to the Canadian test, which are as follows: ladder climbing for a second time, lowering a ladder and carrying it and victim carry. Additionally, in the Dutch test, some parts were added compared to the Canadian test, including: getting ready for turn-out, walking a balance beam, stepping/climbing over a fence, smoke dive simulation with hose, and ceiling demolition simulation. This warrants the need for another study to assess the reliability of the FFST. Moreover, there is another reason to study the test again: the test-retest period was chosen for a longer period in the present study. In the study of Deakin et al. [15], a one-day test-retest period was chosen while in the present study one-week and three-week test-retest periods were studied. Testing moments should be comparable, because it is unwanted to find differences due to change in the physical condition of the fire fighter tested. Testing moments chosen were acceptable within a couple of weeks. Therefore, the test was not only executed with a one-week period, but also with a three-week test-retest period. Advancing statistical science leads to the application of another statistical method in the present study to test the reliability: ICC [18]. Because another statistical method was used, results from Deakin et al. [15] and the present study cannot be compared exactly, even though both studies have similar results; that is, both studies demonstrate good reliability for the functional fire fighting simulation tests.

We also studied the possible influences of the differences in days between the multiple test-retest moments, which included variations between the first and second performances of 5 - 10 days and between the second and third performances of 20 - 28 days. Posthoc studying with a scatterplot showed no trends in performance between shorter or longer in-between times.

### Agreement

The learning effect from the first to the second performance seems to be large considering that the mean testing time shifted from 828 s to 754 s after one week. A trend was seen in which fire fighters improved when performing the test a second time. A reason for this trend might be that fire fighters did not know what to expect when they performed the test initially. Examples of reactions that were often heard from fire fighters after their first execution are as follows: "If I take the test again I would do it differently because I know what to expect". The standard error of measurement (SEM), reflecting the agreement, depends on changed outcomes at the different testing moments within-subjects. The SEM after one week, between the first performance and second performances of the FFST, was 70 s; therefore, we advise that one not make a definitive judgement based on the outcome of the FFST when one performs the test for the first time. This study showed that the SEM became lower (40 s) when comparing test-retest moments after a three-week period (in which participants performed the test at minimum for a second and third time). These SEM values may help with the interpretation of the testing time when within-subject comparisons are made and indicate that administrators of the test should give fire fighters the opportunity to get acquainted with the FFST.

A SEM of 40 s is 5% of the mean testing time of the third performance. If the change is smaller than 40 s, measurement error should be the explanation. Therefore, changes have to be larger than 40 s to assure that observed differences are not due to measurement error.

The difference found in the two ICCs, 0.56 and 0.79, may be explained by the measurement error for the first and second measurement; the reliability depends on variability between the persons and measurement error. If the measurement error is larger, reliability will decrease when the variation is about the same [18]. In our study, measurement error was larger in the first and second measurements, as in the later measurements, while variation was about the same (see SD in Table 2). As a result, the ICC was lower between the first and second compared to the second and third measurements.

### Construct validity

The problem in this study, as in many others, is that there is no gold standard that measures the same construct as the FFST, which is the work ability of fire fighters tested during job-simulated activities. In the case of the absence of a gold standard, Rutjes et al. [21] proposed that a composite reference standard be used. Accordingly, convergent validity, as a part of construct validity, was tested with the construct of combined self-rated and supervisors-rated work ability. However, the problem in these situations is that the composite reference standard is also a new instrument, and it is unknown whether it correctly covers the right construct. With these shortcomings of construct validity in mind, we found moderate-sized correlations to confirm construct validity. In addition, another reason for using the FFST is that, in addition to the results given in this study, an expert meeting was organised in which experts from the fire fighter sector pointed out that the FFST was a reflection of real fire fighting tasks. This positive result of content validity of the FFST argues in favour of application of the FFST in the WHS of Dutch fire fighters.

The implications of the results of the present study are that we recommend the use of the FFST in the WHS for Dutch fire fighters. The final test criterion by which to judge the FFST will be developed in the future within the fire fighting sector, in which it can be imagined that the fire fighter will not be judged by the speed of executing the test alone, but perhaps also by the technical performance. After developing the final test criterion and cut-off points for judging the performance, and after implementation of the FFST, the test criterion should be evaluated after several years.

### Limitations

In an a-priori power analysis, it turned out that we needed 23 subjects for the reliability part of the study (confidence level 0.95 and ICC between 0.9 and 0.8). Due to the fact that the results of only 19 subjects for the test could be used, it is possible that the precision of the results was less and the 95% borders of the ICC confidence interval were broader.

Performances of fire fighters were standardised to daytime performance within the test-retest part of the study. It was not possible to plan the three testing moments for each fire fighter at exactly the same time of the day. This may be seen as a limitation in this study. Nevertheless, we think that at the group level, the influence is random and it therefore did not influence the overall outcome.

## Conclusions

The reliability of the FFST was high at the three-week test-retest period. The agreement reflected by the SEM after three weeks provides evidence that the FFST could be used in practice. A change has to be larger than 40 s to assure that differences are not due to the measurement error. Construct validity of the FFST with ratings of work ability was moderate. We recommend that the FFST be used as a part of the Workers' Health Surveillance for Dutch fire fighters, pending further development of its test criterion. Fire fighters should get acquainted with the test, and we advise that they should perform the FFST once as a trial before judging their performance on testing time during the second performance.

## Competing interests

The authors declare that they have no competing interests.

## Authors' contributions

MP coordinated the study, performed the statistical analysis and drafted the manuscript. MF and JS designed the study, monitored progress and were engaged in drafting the manuscript. MF and JS were co-principal investigators. All authors read and approved the final manuscript.

## Pre-publication history

The pre-publication history for this paper can be accessed here:

http://www.biomedcentral.com/1472-6963/10/32/prepub

## References

[B1] Technical and ethical guidelines for workers' health surveillance (OSH No. 72) 1998 Geneva, International Labour Office (Occupational Safety and Health Series No. 72)

[B2] Sluiter JK Frings-Dresen MH What do we know about ageing at work? Evidence-based fitness for duty and health in fire fighters Ergonomics 2007 50 11 1897 1913 10.1080/00140130701676005 17972208

[B3] Sothmann MS Saupe K Jasenof D Blaney J Heart rate response of firefighters to actual emergencies. Implications for cardiorespiratory fitness J Occup Med 1992 34 8 797 800 10.1097/00043764-199208000-00014 1506937

[B4] Gledhill N Jamnik VK Characterization of the physical demands of firefighting Can J Sport Sci 1992 17 3 207 213 1325260

[B5] Smith DL Petruzzello SJ Kramer JM Misner JE Physiological, psychophysical, and psychological responses of firefighters to firefighting training drills Aviat Space Environ Med 1996 67 11 1063 1068 8908345

[B6] Bos J Mol E Visser B Frings-Dresen M The physical demands upon (Dutch) fire-fighters in relation to the maximum acceptable energetic workload Ergonomics 2004 47 4 446 460 10.1080/00140130310001643283 14681000

[B7] Holmér I Gavhed D Classification of metabolic and respiratory demands in fire fighting activity with extreme workloads Applied ergonomics 2007 38 45 52 10.1016/j.apergo.2006.01.004 16516136

[B8] Lusa S Louhevaara V Kinnunen K Are the job demands on physical work capacity equal for young and aging firefighters? J occup med 1994 36 1 70 74 8138852

[B9] Sluiter JK Frings-Dresen MH Pre-employment and Periodical Workers' Health Surveillance in the Fire fighting sector In Dutch: Aanstellingskeuring en Periodiek Preventief Medisch Onderzoek (PPMO) voor de Brandweersector 2006 Amsterdam: Coronel Institute for Occupational and Environmental Health, Academic Medical Center report no. 06-03

[B10] Sluiter JK High-demand jobs: Age-related diversity in work ability? Applied Ergonomics 2006 37 429 440 10.1016/j.apergo.2006.04.007 16764815

[B11] Sothmann MS Gebhardt DL Baker TA Kastello GM Sheppard VA Performance requirements of physically strenuous occupations: validating, minimum standards for muscular strength and endurance Ergonomics 2004 47 8 864 875 10.1080/00140130410001670372 15204279

[B12] Punakallio A Trial-to-trial reproducibility and test-retest stability of two dynamic balance tests among male firefighters Int J Sports Med 2004 25 3 163 169 10.1055/s-2003-45251 15088238

[B13] Williams-Bell FM Villar R Sharratt MT Hughson RL Physiological demands of the firefighter candidate physical ability test Med Sci Sports Exerc 2009 41 3 653 662 10.1249/MSS.0b013e31818ad117 19204584

[B14] Reilly T Iggleden C Gennser M Tipton M Occupational fitness standards for beach lifeguards. Phase 2: the development of an easily administered fitness test Occup Med 2006 56 1 12 17 10.1093/occmed/kqi168 16267102

[B15] Deakin JM Pelot R Smith JT Stevenson JT Wolfe LA The development of a bona fide physical maintenance standard for CD and DND fire fighters 1998 Ergonomics Research Group Queens University Kingston, Ontario

[B16] van Blitterswijk M Lagrand R Zijden J van der Guideline Task Specific Condition testing, for fire fighters Rotterdam 2006 [In Dutch: Handboek Taak Specifieke Conditietest voor uitrukdienst-personeel Brandweer Rotterdam]. Rotterdam

[B17] de Vet HC Terwee CB Bouter LM Current challenges in clinimetrics Journal of Clinical Epidemiology 2003 56 1137 1141 10.1016/j.jclinepi.2003.08.012 14680660

[B18] de Vet HC Terwee CB Knol DL Bouter LM When to use agreement versus reliability measures Journal of Clinical Epidemiology 2006 59 1033 1039 10.1016/j.jclinepi.2005.10.015 16980142

[B19] Streiner DL Norman GR Reliability Health Measurement scales: a practical guide to their development and use 2003 3 Oxford Medical Publications 126 152

[B20] Streiner DL Norman GR Validity Health Measurement scales: a practical guide to their development and use 2003 3 Oxford Medical Publications 172 193

[B21] Rutjes AW Reitsma JB Coomarasamy A Khan KS Bossuyt PM Evaluation of diagnostic tests when there is no gold standard: A review of methods Health technology assessment 2007 11 50 18021577 10.3310/hta11500

[B22] Thomas S Reading J Shephard RJ Revision of the Physical Activity Readiness Questionnaire (PAR-Q) Can J Sport Sci 1992 17 4 338 345 1330274

[B23] Tuomi K Ilmarinen J Jahkola A Katajarinne L Tulkki A Work Ability Index 1997 Helsinki: Finnish Institute of Occupational Health

[B24] Shrout PE Fleiss JL Intraclass Correlations: Uses in Assessing Rater Reliability Psychological Bulletin 1979 86 2 420 428 10.1037/0033-2909.86.2.420 18839484

[B25] Terwee CB Bot SD de Boer MR Windt DA van der Knol DL Dekker J Bouter LM de Vet HC Quality criteria were proposed for measurement properties of health status questionnaires Journal of Clinical Epidemiology 2007 60 34 42 10.1016/j.jclinepi.2006.03.012 17161752

[B26] Bland JM Altman DG Statistical methods for assessing agreement between two methods of clinical measurement Lancet 1986 1 307 310 2868172

[B27] Innes E Straker L Validity of work-related assessments Work 1999 13 125 152 12441557

